# Patient reported outcomes in genital gender-affirming surgery: the time is now

**DOI:** 10.1186/s41687-022-00446-x

**Published:** 2022-04-25

**Authors:** Nnenaya Agochukwu-Mmonu, Asa Radix, Lee Zhao, Danil Makarov, Rachel Bluebond-Langner, A. Mark Fendrick, Elijah Castle, Carolyn Berry

**Affiliations:** 1 Department of Urology, NYU School of Medicine, New York University, 221 East 41st Street, New York, NY 10017 USA; 2 Department of Population Health, NYU School of Medicine, New York, NY USA; 3 Department of Medicine, NYU School of Medicine, New York, NY USA; 4 Callen-Lorde Community Health Center, New York, NY USA; 5 Department of Plastic and Reconstructive Surgery, NYU School of Medicine, New York, NY USA; 6 Department of Medicine, University of Michigan, Ann Arbor, MI USA; 7 Department of Public Policy, University of Michigan, Ann Arbor, MI USA

**Keywords:** Patient reported outcomes, PROs, Gender-affirming surgery, Vaginoplasty, Metoidioplasty, Phalloplasty

## Abstract

Transgender and non-binary (TGNB) individuals often experience gender dysphoria. TGNB individuals with gender dysphoria may undergo genital gender-affirming surgery including vaginoplasty, phalloplasty, or metoidioplasty so that their genitourinary anatomy is congruent with their experienced gender. Given decreasing social stigma and increasing coverage from private and public payers, there has been a rapid increase in genital gender-affirming surgery in the past few years. As the incidence of genital gender-affirming surgery increases, a concurrent increase in the development and utilization of patient reported outcome measurement tools is critical. To date, there is no systematic way to assess and measure patients’ perspectives on their surgeries nor is there a validated measure to capture patient reported outcomes for TGNB individuals undergoing genital gender-affirming surgery. Without a systematic way to assess and measure patients’ perspectives on their care, there may be fragmentation of care. This fragmentation may result in challenges to ensure patients’ goals are at the forefront of shared- decision making. As we aim to increase access to surgical care for TGNB individuals, it is important to ensure this care is patient-centered and high-quality. The development of patient-reported outcomes for patients undergoing genital gender-affirming surgery is the first step in ensuring high quality patient-centered care. Herein, we discuss the critical need for development of validated patient reported outcome measures for transgender and non-binary patients undergoing genital reconstruction. We also propose a model of patient-engaged patient reported outcome measure development.

## Background

Approximately 1 in every 200 US adults, roughly 1.4 million Americans, identify as transgender [1]. Some transgender and non-binary (TGNB) individuals experience gender dysphoria, which is discomfort, distress, physical, and psychological impairment that results from an incongruence between an individual’s gender identity and their sex assigned at birth [2]. TGNB individuals who experience gender dysphoria may seek medical and/or surgical interventions so that their physical features are congruent with their gender identity. In the past decade increased recognition of gender dysphoria, decreasing social stigma towards TGNB individuals, and increasing insurance coverage have led to a three-fold increase in gender-affirming surgeries [3–6]. Of gender-affirming surgeries, the incidence of genital gender-affirming surgery—vaginoplasty, phalloplasty and metoidioplasty—has steeply increased and is likely the most common inpatient gender-affirming surgery [7]. Although increased coverage has undoubtedly had many benefits for the TGNB community, to date, there have been few, if any, attempts to systematically assess patients’ perspectives on genital gender-affirming surgery. Without direct input from patients undergoing gender-affirming surgery, we cannot truly understand patients’ goals and preferences (e.g., sexual and aesthetic goals, quality of life) beyond amelioration of gender dysphoria, nor can we reliably assess the magnitude of benefits of gender-affirming surgery or prepare patients with realistic expectations of genital surgeries. Perhaps the most impactful result of a lack of explicit capture and incorporation of the patient perspective is the lack of shared decision-making and propagation of a paternalistic care model. This is evidenced by single-center studies, which have demonstrated evidence of decision-related regret and depending on an individuals’ goals, revision surgery [8–10]. There is also evidence that patients’ knowledge about outcomes after gender-affirming surgery is lacking and patients may have unrealistic expectations [11]. The current system of outcome reporting prioritizes clinical outcomes, which only captures physicians’ reports of outcomes, is subject to bias, does not include the patients’ perspective and, hence, are inadequate. The process of genital reconstruction is intensive and patients undertake significant risk to undergo life-changing genital gender-affirming surgery; there is an urgent need for patient-centered metrics. Patient reported outcome measures (PROMs) are patient-centered metrics and represent a viable solution to these challenges and shortcomings.

## Main text

PROMs developed *by and for* TGNB patients undergoing genital gender-affirming surgery are imperative to delivering high-value, high-quality, patient-centered care. There has been an increased recognition of the importance of PROMs generally, with concurrent emphasis on the patient experience as a fundamental component of quality of care. PROMs as defined by the FDA are “measurement[s] based on a report that comes directly from the patient about the status of a patient’s health condition without amendment or interpretation of the patient’s response by a clinician or anyone else.” [12] PROMs are patient-generated and patient-centered health data, are measures of care delivery, evaluate patients’ symptoms, functional status, health related quality of life, satisfaction with care, and provide a holistic view of the patient experience [13, 14]. While PROMs have traditionally been used as research tools, they are now recognized as meaningful clinical data elements, which may in certain instances be more accurate than those assessed by clinicians [15, 16]. PROMs have been shown to support clinical improvements and positively impact patients in several fields [14, 17–21]. In addition, preliminary data has demonstrated that PROMs may have an overwhelmingly positive impact in gender-affirming surgery as well [22]. Moreover, the TGNB community desires high-quality, long-term outcome data [23]. PROMs are especially necessary in reconstructive surgery given the challenge in evaluating short and long-term outcomes and quality. Reconstructive surgery is a complex journey for a patient and is purely patient-driven; PROMs will ensure that this journey is patient- centered at each step including the initial consultation, decision-making process, surgery, and perhaps most importantly, outcome reporting and measurement.

While wide agreement for the need for PROMs in gender-affirming care exists [24, 25], there are many challenges to their development and implementation [26]. Questions such as how data should be most effectively collected, visualized, shared, and used to improve quality have limited the routine use of PROMs in clinical care [21]. Surmounting these challenges begins by considering the benefit of PROMs at the patient, provider, and system levels [27]. At the patient level, PROMs can help patients undergoing genital gender-affirming surgery develop realistic expectations. In addition, PROMs provide an opportunity to understand patients’ priorities and enable them to become fully informed about benefits, risks, and available options much earlier in the process of seeking genital gender-affirming surgery. The routine collection of PROMs for patients undergoing genital gender-affirming surgery and their utilization in clinical practice can facilitate the provision of a roadmap for patients at each step on this journey. Ultimately, counseling with the use of PROMs can inform patients’ decision regarding whether to have surgery and which surgery to have (e.g., metoidioplasty vs. phalloplasty).

At the provider level, there is evidence that PROMs improve patient and physician satisfaction, increase workflow efficiency, and enable critical discussions [28]. PROMs help enhance both patient and provider satisfaction by helping physicians set appropriate expectations regarding patients’ outcomes. PROMs can also improve relationships and communication between physicians and patients as surgeons gain better understanding of patients’ priorities and desired outcomes from surgery [28]. The availability and utilization of PROMs may greatly influence the success of surgery and potentially avoid the need for revision surgery, which is beneficial to the patient and the provider. The success of surgery is highly dependent on an individual patient’s values and preferences; a physicians’ definition of success may be highly divergent from a patients’ definition. PROMs magnify the individuals’ voice and thereby emphasize and facilitate, for the provider, a patient- centered model of care. This patient-centered model of care, facilitated in part by PROMs, portends higher chances of success for the patient. It also gives the surgeon and team an opportunity to understand what is important to their patients. The use of a PROM tool in this context may be a segue to the development and use of tools to measure patient reported experience measures as well, which would further improve patients’ experience of gender-affirming surgery [29].

Finally, at the system level, one can use PROMs as a quality metric [26]. Distinct from clinical outcomes, PROMs can be used to measure structures, processes, and outcomes of health care and thereby, can improve quality of care at each step and in several ways [30]. PROM data can be used to evaluate variation in patient care—specifically, variation in the “best” outcomes from the patient’s perspectives and subsequently, areas for quality improvement. This can thereby lead to modified *processes* to improve *outcomes* and quality. PROMs focus on the effectiveness and experience of care, both of which are essential components of quality. Incorporating PROMs facilitates shared decision-making, which also improves quality of care. Additionally, PROMs have the unique ability to capture two additional major components of clinical care provision and quality—provider accountability and performance measurement. The potential of collaborative quality improvement among providers in this setting is vast. At the policy level, PROMs are fundamental for a transition from volume to value-based healthcare reform. Validated PROMs may also contribute to advocacy efforts for wider coverage and policies that serve transgender and non-binary patients. In this context, validation is important and refers to a PROM tool, which measures what it intends to measure in the target population [12]. PROMs have been and are currently used in genital gender-affirming surgery research, though they are not validated for use in transgender and non-binary populations nor are they specific for genital gender-affirming surgery [25].

**Fig. 1:**
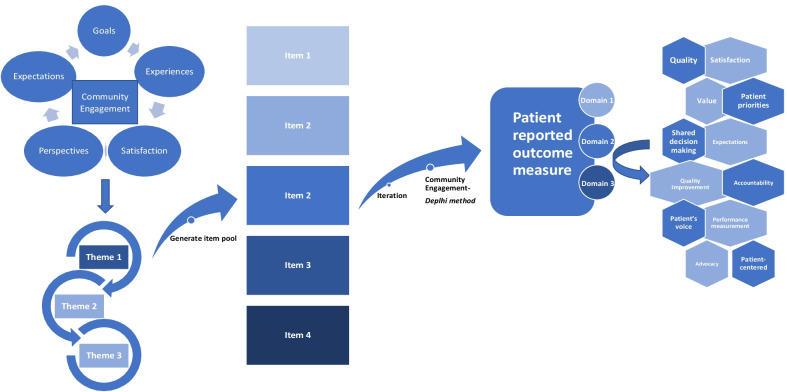
Patient-engaged conceptual model of patient reported outcome measure development

**Fig. 2:**
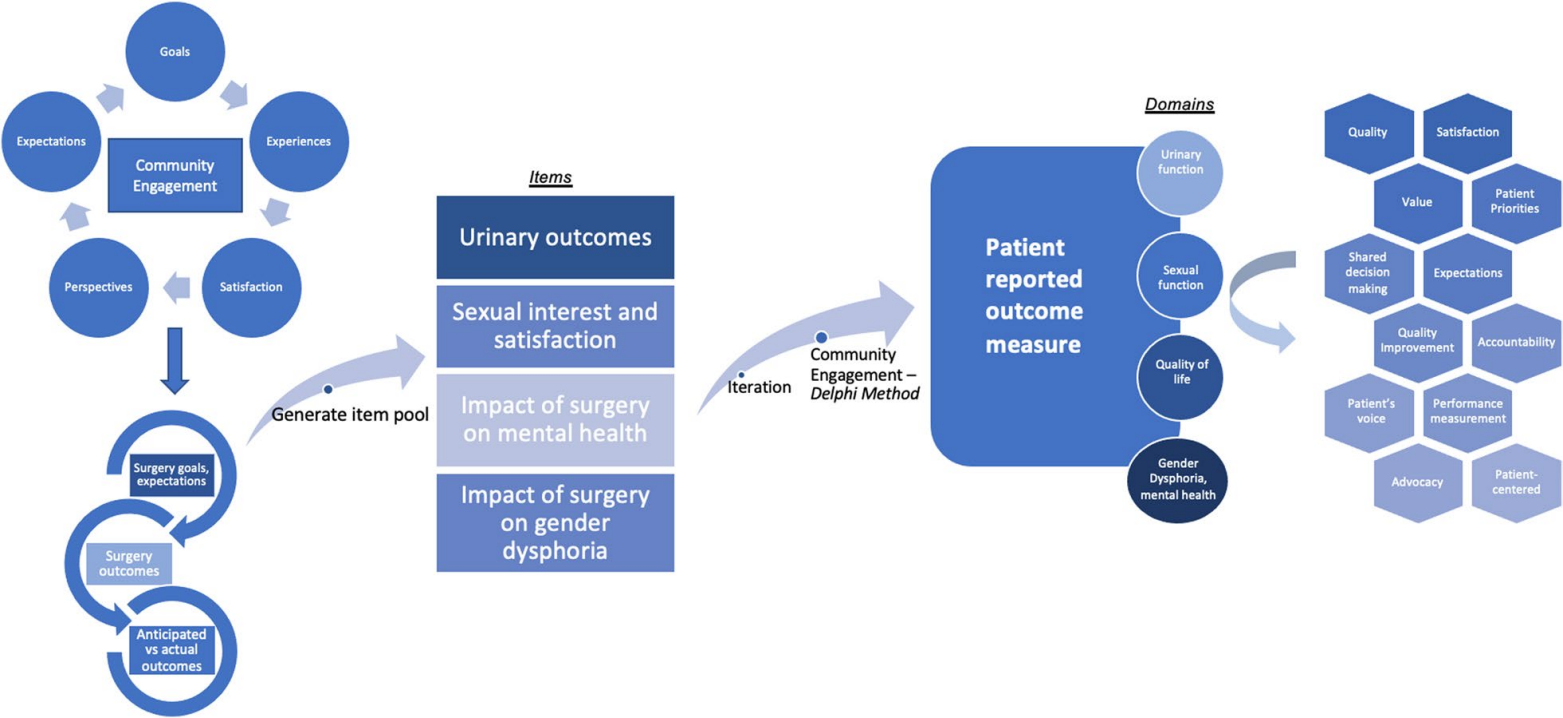
Potential themes, items, and domains resulting from community-based participatory research model of PROM tool development in genital gender-affirming surgery

The primary role of PROMs is to capture the patients’ voice; given this, amplification of the patient’s voice at the forefront of PROM development is crucial. Figure 1 demonstrates a conceptual model for patient- engagement in PROM development; it is critical to have a conceptual framework to guide PRO measurement and assessment [31]. We plan to magnify the patients’ voices at each stage of development of PROMs for genital gender-affirming surgery. We propose early engagement with members of the transgender and non-binary communities in a Community-Based Participatory Research model. Engagement and collaboration with the TGNB community is fundamental in development of PROMs that are meaningful and relevant to the TGNB community and most importantly it will illuminate that which is often invisible—the patient perspective. Through engagement with LGBTQ community health centers, we are conducting focus group sessions for patients who will undergo or who have undergone phalloplasty, vaginoplasty, and metoidioplasty to gain a deep and detailed understanding of goals, experiences, quality of life, expectations, and aspirations. Qualitative analysis will then lead to convergent themes as depicted in Fig. 2. Members of the transgender and non-binary community are leading or co-leading the focus group sessions. From initial discussions, dialogue, and thematic analysis, we will generate a pool of potential items for a PROM tool. This tool will then be rigorously vetted by the transgender and non-binary community members via a modified Delphi method, an iterative process whereby through data collection, analysis, and interpretation, the item pool is further modified with repeat in the cycle until there is agreement [32]. This enables and prioritizes PROMs that align with actual patient outcomes; this is divergent from the current outcome measurements, which prioritize surgical opinion. Only once agreement and homogeneity are reached amongst experts including TGNB focus group participants, TGNB community partners, qualitative researchers, and genital gender-affirming surgeons will decisions be made on items for the final tool, which would encompass domains (e.g., sexual, urinary, quality of life). This process will refine and finalize the tool which will then undergo rigorous validation testing. The final component of Figs. 1 and 2 depict the potential outcomes and areas of improvement from utilization of this PROM tool, including quality improvement, satisfaction, value, and shared decision making.

## Conclusion

PROMs for genital gender-affirming surgery are long overdue. Through intensive community engagement, we aim to develop PROMs with the transgender and non-binary community and inform the process of patient-centered care to serve transgender and non-binary patients. If surgeons who provide essential gender-affirming surgical care embrace the opportunity to be early adopters of PROMs, we can transform patient- centered care by making it a reality for transgender and non-binary patients.

## Data Availability

Not applicable.

## Abbreviations

PRO: Patient reported outcomes
TGNB: Transgender and non-binary
